# Incidence of hepatitis C virus infection among people living with HIV: An Egyptian cohort study

**DOI:** 10.4102/sajhivmed.v23i1.1442

**Published:** 2022-11-09

**Authors:** Fatma Elrashdy, Suzan Hagag, Rahma Mohamed, Shereen Abdel Alem, Safa Meshaal, Ahmed Cordie, Aisha Elsharkawy, Gamal Esmat

**Affiliations:** 1 Department of Endemic Medicine and Hepatology, Faculty of Medicine, Cairo University, Cairo, Egypt; 2 Department of Public Health and Community Medicine, Faculty of Medicine, Cairo University, Cairo, Egypt; 3 Kasr Al-Aini HIV and Viral Hepatitis Fighting Group, Cairo University Hospitals, Cairo University, Cairo, Egypt; 4 Department of Clinical Pathology, Faculty of Medicine, Cairo University, Cairo, Egypt

**Keywords:** incidence rate of HCV, HCV screening, people living with HIV, Egypt, IDU, HCV seroconversion

## Abstract

**Background:**

Egypt used to have one of the highest hepatitis C virus (HCV) infection prevalence rates worldwide, with an estimated HCV prevalence of around 4.5% to 6.7%.

**Objectives:**

To determine the HCV infection incidence rate amid Egyptian patients living with HIV.

**Method:**

A total of 460 HIV-positive patients were recruited in a retrospective cohort study from Imbaba Fever Hospital, Cairo, between January 2016 and March 2019. The patients had a negative baseline and at least one other HCV antibody test. Hepatitis C virus antibody testing was done by antibody sandwich third-generation enzyme-linked immunosorbent assay. The hepatitis C virus infection incidence rate among HIV-infected patients was calculated using the person-time incidence rate.

**Results:**

Two hundred and eighteen patients were finally included: 146 (31.7%) patients were excluded for having a positive baseline HCV Ab result and 96 patients were excluded for not having a follow-up HCV Ab test. Eighteen patients had HCV seroconversion (8.3%), achieving an incidence rate of 4.06 cases per 100 person-years (95% confidence interval: 3.87–4.24). Injection drug use (IDU) was the commonest risk factor among seroconverters, with an HCV incidence rate of 7.08 cases per 100 person-years. Injection drug use history was reported in 83.3% of the seroconverters and in only 47.2% of non-seroconverters; *P* = 0.005.

**Conclusion:**

Egyptian HIV-infected patients show a high incidence rate of HCV infection especially among those who have a history of IDU. Accordingly, attention should be paid for prevention, screening and timely treatment of HCV in patients infected with HIV.

**What this study adds:**

The demonstration of a high HCV infection incidence rate among HIV-infected patients and shows the need for screening and prevention in this population.

## Introduction

The hepatitis C virus (HCV) and HIV infections, which share some of the same modes of transmission and affected populations,^1^ represent major public health problems worldwide. Globally, there are 58 million people with chronic HCV infection, 37.7 million people infected with HIV and 2.3 million with HIV/HCV co-infection.^1,2,3^

Egypt used to have one of the highest HCV infection prevalence rates worldwide, with an estimated HCV prevalence of 4.5% to 6.7%.^4^ Upon introduction of tolerable oral direct acting antiretrovirals (DAAs) in 2014 and the start of an unprecedented nationwide HCV screening and treatment campaign in Egypt in 2018 to achieve the World Health Organization (WHO) 2030 HCV disease elimination target, the prevalence of HCV declined dramatically; it is expected to reach less than 0.5% during 2020.^5,6,7^

However, special high-risk groups of HCV-infected patients like people who inject drugs (PWID) and people living with HIV (PLHIV) need more attention and micro-elimination strategies to achieve the WHO elimination goal.^8^

HIV/HCV co-infection changes the course and outcomes of both infections significantly.^9^ Chronic HCV infection increases morbidity and mortality among PLHIV.^10^ Hepatitis C virus causes chronic inflammation with impaired immune system reconstitution after anti-retroviral therapy (ART).^9,11^ In addition, HCV increases the risk of renal, cardiovascular diseases and hepatocellular carcinoma (HCC).^9^ People living with HIV have a higher HCV viral load with less possibility of spontaneous clearance of HCV infection.^12^ Moreover, HIV makes HCV a more aggressive infection with accelerated progression to liver cirrhosis, liver cell failure and HCC.^13^

In 2020, the Joint United Nations Programme on HIV/AIDS (UNAIDS) stated that out of the Middle East and North Africa region (MENA), Egypt has the fastest growing HIV epidemic despite low (< 0.1% by the end of 2020) HIV prevalence in the general population.^14^ Over the past 10 years, incidence of HIV increased by 25% – 35% every year with men who have sex with men (MSM) and PWID being the most affected groups.^14^

There are scarce data about the burden of and risk factors for HCV infection among PLHIV in Egypt. Therefore, the aim of this study is to fill the existing knowledge gap on HCV epidemiology among patients infected with HIV attending one of the large reference centres of HIV in Cairo, Egypt.

## Methods

### Study participants and settings

We performed a retrospective cohort study in which we recruited PLHIV aged 18 years or older attending Imbaba Fever Hospital, Cairo, between January 2016 and March 2019, excluding patients who refused to participate. The inclusion criteria for this study was a negative baseline anti-HCV test and at least one subsequent HCV antibody test during the study time.

### Study methods

Demographic data were reported including age, gender and marital status. Data about self-reported risk factors for HIV infection were collected as history of injection drug use (IDU), risky sexual behaviour, previous operations and dental procedures. The status of HIV was assessed regarding the current CD4 T-cell count, plasma HIV viral load and ART treatment status.

Hepatitis C virus antibody testing was done by using the Murex anti-HCV third-generation enzyme-linked immunosorbent assay (ELISA) (version 4) sandwich technique (Abbott Laboratories, Abbott Park, Illinois, United States). The first positive anti-HCV test during follow-up was considered as the HCV seroconversion. The midpoint between the first positive and last negative anti-HCV tests was estimated as the date of this seroconversion (incident HCV infection).

The person-time incidence rate is defined as the number of new cases of a disease occurring in a population during a specified period of time per total person-time (sum of the time period of observation of each person who has been observed). The equation we used to calculate HCV incidence rate was:

$$\frac{\text{number of HCV seroconversion events}}{\text{person-time at risk}}$$ [Eqn 1]

Observation durations were calculated until HCV seroconversion, death, loss to follow-up or the end of the study, whichever occurred first.

### Data management

Data were analysed by mean and standard deviation in the case of quantitative data, and by frequencies (number of cases) and percentages in the case of categorical data. A Chi-square test was used to compare categorical data. *P*-values less than 0.05 were considered statistically significant. A Mann–Whitney U test was used to compare quantitative variables between the studied groups. When the expected frequency was less than 5, we used an exact test instead. All data were processed using IBM^®^ SPSS^®^ version 22 (IBM Corp., Armonk, New York, United States).

### Ethical considerations

The study was designed to respect all ethical guidelines of the 1975 Declaration of Helsinki and was approved by the Institutional Review Board of the Faculty of Medicine, Cairo University (approval code N-149-2018).

## Results

### Incidence rate of hepatitis C virus infection

**FIGURE 1:**
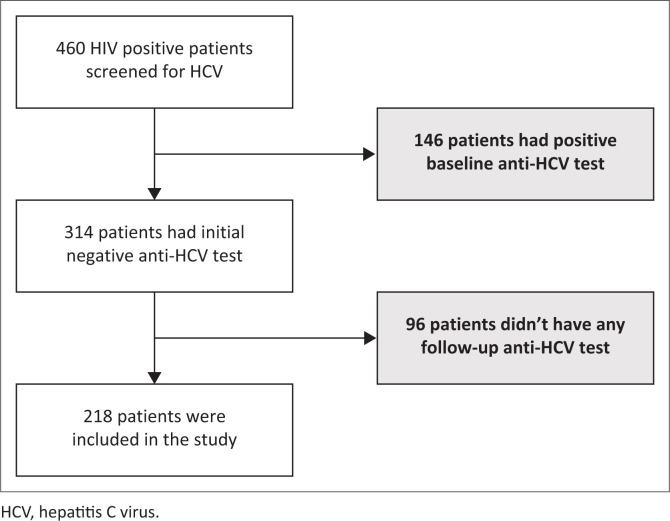
A flowchart illustrating the study population selection. Grey boxes denote the patients excluded from the study.

Four hundred and sixty patients were screened. After exclusion of patients with a positive baseline HCV test and who didn’t have any follow-up anti-HCV test, 218 patients were finally included (Figure 1). The 218 patients at risk of incident HCV infection at baseline contributed 5312 person-months or 443 person-years of observation. Eighteen patients had HCV seroconversion representing an incidence rate of 4.06 per 100 person-years (95% confidence interval [CI]: 3.87–4.24).

### Characteristics of patients with and without hepatitis C virus seroconversion

At the time of seroconversion the mean age was 42.3 years, and 83.3% of seroconverters were male. No significant difference was found between HCV Ab negative patients and HCV seroconverters (Table 1).

**TABLE 1:** Sociodemographic characteristics and HIV status of study participants.

Characteristic	Total (*n* = 218)			HCV Ab negative (*n* = 200; 91.7%)			HCV seroconverters (*n* = 18; 8.3%)			*P*
	*n*	%	Mean ± s.d.	*n*	%	Mean ± s.d.	*n*	%	Mean ± s.d.	
**Age (year)**	-	-	41.6 ± 10.6	-	-	41.6 ± 10.8	-	-	42.3 ± 8.9	NS
**Gender**										
Male	147	67.4	-	132	66.0	-	15	83.3	-	NS
Female	71	32.6	-	68	34.0	-	3	16.7	-	
**Marital status**										
Single	111	50.9	-	100	50.0	-	11	61.1	-	NS
Married	107	49.1	-	100	50.0	-	7	38.9	-	
**CD4 cell count at initial visit**	-	-	398 ± 92	-	-	398 ± 191	-	-	396 ± 199	NS
**Mean HIV RNA at initial visit (copies/mL)**	33 668	-	-	30 610	-	-	67 139	-	-	NS
**ART status**										
Never used ART	30	13.8	-	27	13.5	-	3	16.7	-	NS
On treatment	188	86.2	-	173	86.5	-	15	83.3	-	

HCV, hepatitis C virus; NS, non-significant; ART, antiretroviral treatment; s.d., standard deviation.

Injection drug use (*P*-value 0.005), history of dental procedures (*P*-value < 0.001) and previous operations (*P*-value 0.019) were significantly higher in HCV seroconverters compared to HCV Ab negative patients (Table 2).

**TABLE 2:** Self-reported risk factors for HIV and hepatitis C virus among study participants.

Characteristic	Total (*n* = 218)		HCV Ab negative (*n* = 200; 91.7%)		HCV seroconverters (*n* = 18; 8.3%)		*P*
	*n*	%	*n*	%	*n*	%	
**Risky sexual behaviour**							
Yes	4	1.9	3	1.6	1	5.6	NS
No	207	98.1	190	98.4	17	94.4	
**Intravenous drug use history**							
Yes	108	50.2	93	47.2	15	83.3	0.005
No	107	49.8	104	52.8	3	16.7	
**Dental procedure**							
Yes	27	12.4	17	8.5	10	55.6	< 0.001
No	191	87.6	183	91.5	8	44.4	
**Previous operation**							
Yes	14	6.4	10	5	4	22.2	0.019
No	204	93.6	190	95	14	77.8	

HCV, hepatitis C virus; NS, non-significant.

There were 108 PWID at risk of HCV infection at baseline contributing 2541 person-months or 212 person-years of observation. Fifteen of the 108 PWID had HCV seroconversion representing an HCV incidence rate of 7.08 per 100 person-years (95% CI: 6.72–7.44).

## Discussion

In this cohort of Egyptian PLHIV, the HCV infection incidence rate is 4.06 per 100 person-years. This high HCV incidence rate among Egyptian PLHIV is likely due to several factors. First, there is a lack of effective prevention programmes for HIV and HCV transmission in Egypt like opioid substitution therapy (OST) and needle and syringe programmes (NSP).^15^ Second, there is limited HCV screening in some high-risk groups like MSM, despite the well-established screening programme dedicated to PWID as a part of the process to meet WHO targets of HCV elimination.^16^ Third, there are barriers of access to treatment of HCV in PLHIV, like fear of community stigma.^17^

The incidence rate we observed is higher than that of the incidence of HCV infection in the general Egyptian population, which was estimated as 0.08–1.02 per 100 person-years by a systematic review conducted in 2018.^18^ Moreover, in 2020, prevalence of HCV infection in the general Egyptian population is expected to decrease to less than 0.5%, thanks to the nationwide screening and treatment programme ‘100 Million Health’ started in 2018.^5^ However, the HCV infection incidence rate among Egyptian PLHIV remains high, raising the need for more effective application of prevention programmes, regular screening and micro-elimination strategies of HCV in PLHIV in Egypt.

The available Egyptian and African studies in literature usually reported the epidemiology of HIV/HCV co-infection in terms of prevalence. However, the incidence rate estimation is also needed to assess the rate of new HCV infections among PLHIV so we can better judge the prevention programmes and HCV treatment strategies among this population.

The incidence rates of HIV/HCV co-infection in some Asian and European countries were lower than that reported in this study with a rate of 0.88 per 100 person-years in Singapore,^19^ and 0.72 and 0.44 per 100 person-years among MSM in France^20^ and Italy^21^. A systematic review conducted between 2000 and 2016 reported an incidence rate of 0.78 per 100 person-years among MSM.^22^

In our study, the risk factors found to be associated with getting HCV infection in PLHIV are IDU, history of dental procedures and operations. Injection drug use was associated with the highest incidence rate of HCV infection of 7.08 per 100 person-years.

Globally, the two most vulnerable groups for HIV infection and HIV/HCV co-infection are MSM and PWID.^3^ However, PWID is the commonest group to get these infections in MENA countries because of the religious and social backgrounds that prohibit and limit MSM actions.^1^

In a global systematic review conducted between 01 January 2002 and 28 January 2015, 1.4 million out of 2.3 million HIV/HCV co-infected patients were estimated to be PWID worldwide. The prevalence of HIV/HCV co-infection was estimated as 2.4% in the general population, 6.4% in MSM (highest in North American region) and 82.4% in PWID (highest in MENA region).^1^ In the EuroSIDA cohort study, 23 309 PLHIV were enrolled from the WHO European region and Argentina, showing that 57.4% of patients with HIV/HCV co-infection were PWID.^23^

The effect of HCV infection on CD4 cell count is controversial. Some studies found a decrease in CD4 cell count with HIV/HCV co-infection, hypothesised to be due to the apoptosis of CD4 cells caused by HCV.^24^ Other studies found no effect, like we reported.^25,26^

Our study has some limitations. First, we did not confirm HCV infection by doing HCV polymerase chain reaction, which could have resulted in an over-estimation of incidence. However, a positive HCV antibody test can indicate current infection. Second, most of included patients performed follow-up HCV antibody tests only once. This occurred because of the retrospective nature of the study.

## Conclusion

Despite the great success of HCV infection control in the general Egyptian population thanks to the enormous nationwide screening programme ‘100 Million Health’, the incidence rate of HCV infection in Egyptian PLHIV is high, especially among PWID. Accordingly, more effort is needed in preventive measures of risk factors, regular screening of PLHIV for HCV, and the start of DAA treatment once diagnosed.

## References

[1] Platt L, Easterbrook P, Gower E, et al. Prevalence and burden of HCV co-infection in people living with HIV: A global systematic review and meta-analysis. Lancet Infect Dis. 2016;16(7):797–808. 10.1016/S1473-3099(15)00485-5 26922272

[2] World Health Organization. Hepatitis C: Fact sheet [homepage on the Internet]. [updated 2021 Jul; cited 2022 Mar 18]. Available from: https://www.who.int/en/news-room/fact-sheets/detail/hepatitis-c

[3] UNAIDS. Global HIV & AIDS statistics – 2021 fact sheet [homepage on the Internet]. [cited 2022 Mar 18]. Available from: www.unaids.org/sites/default/files/media_asset/UNAIDS_FactSheet_en.pdf

[4] El-Zanaty and Associates. Egypt health issues survey 2015. Rockville, MD: Ministry of Health and Population, ICF International; 2015.

[5] Waked I, Esmat G, Elsharkawy A, et al. Screening and treatment program to eliminate hepatitis C in Egypt. N Engl J Med. 2020;382(12):1166–1174. 10.1056/NEJMsr1912628 32187475

[6] World Health Organization. Global health sector strategy on viral hepatitis 2016–2021 [homepage on the Internet]. 2016 [cited 2022 Mar 18]. Available from: https://www.who.int/publications/i/item/WHO-HIV-2016.06

[7] Falade-Nwulia O, Suarez-Cuervo C, Nelson DR, et al. Oral direct-acting agent therapy for hepatitis C virus infection: A systematic review. Ann Intern Med. 2017;166(9):637–648. 10.7326/M16-2575 28319996 PMC5486987

[8] Hollande C, Parlati L, Pol S. Micro-elimination of hepatitis C virus. Liver Int. 2020;40(Suppl. 1):67–71. 10.1111/liv.14363 32077601

[9] Soriano V, Vispo E, Fernandez-Montero JV, et al. Update on HIV/HCV coinfection. Curr HIV/AIDS Rep. 2013;10(3):226–234. 10.1007/s11904-013-0169-5 23832718

[10] Collins S, Mertenskoetter T, Loeliger E, et al. Liver-related deaths in persons infected with the human immunodeficiency virus: The D:A:D study. Archiv Intern Med. 2006;166(15):1632–1641. 10.1001/archinte.166.15.1632 16908797

[11] Kim AY, Onofrey S, Church DR. An epidemiologic update on hepatitis C infection in persons living with or at risk of HIV infection. J Infect Dis. 2013;207(suppl_1):S1–S6. 10.1093/infdis/jis927 23390299 PMC3565593

[12] Sulkowski MS. Viral hepatitis and HIV coinfection. J Hepatol. 2008;48(2):353–367. 10.1016/j.jhep.2007.11.009 18155314

[13] Holmberg S, Ly K, Xing J, et al. The growing burden of mortality associated with viral hepatitis in the United States, 1999–2007. Hepatology. 2011;54(4):243.

[14] UNAIDS. Country factsheets: Egypt 2020 [homepage on the Internet]. [cited 2022 Mar 18]. Available from: https://www.unaids.org/en/regionscountries/countries/egypt

[15] Oraby D. Harm reduction approach in Egypt: The insight of injecting drug users. Harm Reduct J. 2013;10:17. 10.1186/1477-7517-10-17 24083418 PMC3849860

[16] World Health Organization. Implementation tool for pre-exposure prophylaxis (PrEP) of HIV infection. Module 10: Testing providers [homepage on the Internet]. 2017 [cited 2022 Apr 11]. Available from: https://apps.who.int/iris/handle/10665/258516

[17] Eletreby R, Esmat G, Elsharkawy A, et al. HCV/HIV coinfected Egyptian patients: A cross-sectional study of their main characteristics and barriers to HCV treatment initiation. Trans R Soc Trop Med Hyg. 2022;116(3):227–232. 10.1093/trstmh/trab106 34291286

[18] Kouyoumjian SP, Chemaitelly H, Abu-Raddad LJ. Characterizing hepatitis C virus epidemiology in Egypt: Systematic reviews, meta-analyses, and meta-regressions. Sci Rep. 2018;8(1):1661. 10.1038/s41598-017-17936-4 29374178 PMC5785953

[19] Ang LW, Choy CY, Ng OT, et al. Hepatitis C virus infection in HIV-infected men in Singapore, 2006–2018: Incidence and associated factors. Sex Health. 2021;18(3):221–231. 10.1071/SH20197 34148565

[20] Castry M, Cousien A, Bellet J, et al. Hepatitis C virus (HCV) incidence among men who have sex with men (MSM) living with HIV: Results from the French Hospital Database on HIV (ANRS CO4-FHDH) cohort study, 2014 to 2017. Euro Surveill. 2021;26(38):2001321. 10.2807/1560-7917.ES.2021.26.38.2001321 34558403 PMC8462035

[21] Cuomo G, Digaetano M, Menozzi M, et al. Incidence of HCV infection amongst HIV positive men who had sex with men and prevalence data from patients followed at the Infectious Diseases Clinic of Modena, Italy. Dig Liver Dis. 2018;50(12):1334–1338. 10.1016/j.dld.2018.05.021 29929780

[22] Ghisla V, Scherrer AU, Nicca D, et al. Incidence of hepatitis C in HIV positive and negative men who have sex with men 2000–2016: A systematic review and meta-analysis. Infection. 2017;45(3):309–321. 10.1007/s15010-016-0975-y 28005195

[23] Fursa O, Mocroft A, Lazarus JV, et al. The hepatitis C cascade of care in HIV/hepatitis C virus coinfected individuals in Europe: Regional and intra-regional differences. AIDS. 2022;36(3):423–435. 10.1097/QAD.0000000000003112 34690281

[24] Potter M, Odueyungbo A, Yang H, et al. Impact of hepatitis C viral replication on CD4+ T-lymphocyte progression in HIV-HCV coinfection before and after antiretroviral therapy. AIDS. 2010;24(12):1857–1865. 10.1097/QAD.0b013e32833adbb5 20479633

[25] Shmagel KV, Saidakova EV, Korolevskaya LB, et al. Influence of hepatitis C virus coinfection on CD4+ T cells of HIV-infected patients receiving HAART. AIDS. 2014;28(16):2381–2388. 10.1097/QAD.0000000000000418 25111083

[26] Korner C, Kramer B, Schulte D, et al. Effects of HCV co-infection on apoptosis of CD4 T-cells in HIV positive patients. Clin Sci. 2009;116(12):861–870. 10.1042/CS20080532 19128241

